# Clinical manifestations, diagnostic criteria, and treatment outcomes of minocycline-associated DRESS syndrome: a comprehensive exploration of published cases

**DOI:** 10.3389/fphar.2025.1515000

**Published:** 2025-07-22

**Authors:** Yan Pan, Qiquan Wu

**Affiliations:** ^1^ Department of Pharmacy, Fuda Cancer Hospital, Guangzhou, Guangdong, China; ^2^ Department of Pharmacy, Ganzhou People’s Hospital, Ganzhou, China

**Keywords:** minocycline, eosinophilia, hypersensitivity, dress, adverse event

## Abstract

**Introduction:**

Minocycline can induce a rare but serious adverse drug reaction known as drug reaction with eosinophilia and systemic symptoms (DRESS) syndrome. We explored the clinical features of minocycline-associated DRESS to aid in early diagnosis and risk mitigation.

**Methods:**

A comprehensive exploration of published cases from the start of electronic databases (PubMed, Embase, Web of Science, CNKI, Wanfang Data) to December 1, 2024. Cases were screened using RegiSCAR criteria.

**Results:**

A total of 3,928 citations were identified through database searches, and after screening, 39 case reports (comprising 57 patients) were included. Fifty-seven patients (mean age 37.1 years) exhibited median DRESS onset at 17.5 days. Respiratory symptoms (87.7%, n = 50) dominated, including non-productive cough, dyspnea, pharyngitis, and wheezing. Fever occurred in 45 (78.9%) patients. Median eosinophilia peaked at 4.09 × 10^9^/L. Symptom improvement median time was 10.5 days. Overall, forty-five patients (93.8% of 48 with outcome data) recovered post-minocycline discontinuation. Four deaths occurred (hepatic failure, refractory hypotension, unknown causes).

**Conclusion:**

Minocycline-associated DRESS syndrome is characterized by diverse clinical manifestations, including prominent respiratory symptoms. Timely drug cessation, corticosteroid therapy, and vigilant monitoring are critical to optimize outcomes. These findings underscore the need for enhanced pharmacovigilance in high-risk populations.

## Introduction

Minocycline is a semi-synthetic tetracycline antimicrobial drug that binds to the A position of the bacterial ribosome 30S subunit, preventing the elongation of the peptide chain and inhibiting the protein synthesis of bacteria or other pathogenic microorganisms, thereby exerting an antibacterial or bactericidal effect (Allen, 1976; Hellmann-Regen et al., 2022). The antibacterial spectrum of minocycline is similar to that of tetracycline, and it is effective against Gram-positive bacteria (including *Staphylococcus aureus*, *Streptococcus*, and *Pneumococcus*), Gram-negative bacteria (including *Neisseria gonorrhoeae*, *Shigella dysenteriae*, *Escherichia coli*, *Klebsiella*), *Treponema pallidum*, *Chlamydia trachomatis*, and *Ureaplasma urealyticum* (Asadi et al., 2020; Yang et al., 2022; Wu et al., 2021; Mojica et al., 2023). While *in vitro* activity has been reported against *Proteus* and *Pseudomonas aeruginosa*, minocycline is not considered clinically effective for infections caused by these organisms. Recent evidence highlights its classification as an antimicrobial agent implicated in antimicrobial-associated DRESS syndrome, particularly through mechanisms involving drug metabolism and immune dysregulation (Sharifzadeh et al., 2021). Data collected from the regulatory approval of minocycline showed that minocycline was generally well tolerated, with the most common adverse events including gastrointestinal symptoms (e.g., abdominal pain, nausea, anorexia, and gastrointestinal disorders) and dizziness (Chow et al., 1975; Martins et al., 2021; Garner et al., 2000). Twenty-nine previous randomized controlled trials (RCTs) reported adverse events attributed to minocycline treatment: 332 of 1906 treated participants (17.4%) experienced one or more adverse events (Garner et al., 2000). Systemic use of minocycline is associated with some serious autoimmune adverse events, including shock and allergic reactions, worsening of systemic lupus erythematosus-like symptoms, nodular arteritis, autoimmune hepatitis, toxic epidermal necrolysis, idiopathic intracranial hypertension, and eosinophilia and systemic symptoms (DRESS) syndrome (Hung and Chung, 2022; Dominic, 2021; Elkayam et al., 1999). Recent studies highlight that systemic use of minocycline is associated with severe hypersensitivity reactions, including DRESS syndrome, which manifests not only as cutaneous eruptions but also profound visceral organ involvement. Pulmonary manifestations (e.g., interstitial pneumonia, pleural effusion, and acute respiratory distress syndrome) occur in up to 50% of cases (Chen et al., 2013), while renal impairment (e.g., acute tubular necrosis, interstitial nephritis) has been reported in 20%–30% of patients (Mori et al., 2019). Cardiac involvement, though less frequent, includes myocarditis and pericardial effusion, contributing to increased mortality risk (Kardaun et al., 2013).

DRESS is a rare but serious adverse event. Rash is commonly observed in DRESS and can progress to involve most parts of the body. It may be associated with systemic involvement of major organs such as the liver or kidneys, leading to elevated liver enzymes, renal dysfunction, and other inflammatory manifestations. Hepatic involvement, manifested by hepatitis, cholestasis, or even fulminant liver failure, represents one of the most frequent and potentially severe organ manifestations. Severe cases may lead to death (Krantz and Phillips, 2023; Hama et al., 2022). Existing studies have shown that DRESS is typically drug-induced, with common causative agents including anti-epileptic drugs (such as lamotrigine, phenytoin, carbamazepine) and antimicrobial drugs (such as vancomycin, trimethoprim-sulfamethoxazole). Studies have shown that minocycline is associated with DRESS (Cardones, 2020; Shiohara and Mizukawa, 2019; Wei et al., 2024), but most of the research on eosinophilia and DRESS is based on case reports, and there is limited understanding of its clinical features and prognosis (Kardaun et al., 2013; Brüggen et al., 2024). The purpose of this study is to explore the clinical characteristics of minocycline-related DRESS. By identifying early diagnostic indicators and understanding the typical progression of the syndrome, clinicians can more promptly recognize and manage DRESS.

## Methods

### Ethical approval and patient consent statements

The study did not require approval from the institutional ethics committee because it was a retrospective analysis of case reports published in a public database and did not involve sensitive personal information.

### Search strategy

Both international (PubMed, Embase, Web of Science) and Chinese (China National Knowledge Infrastructure, Wanfang data) databases were searched independently from their inception to 1 December 2024 with no restriction on the search language. We identified the literature regarding minocycline-induced DRESS via the search terms ‘minocycline’ AND ‘eosinophilia’ OR ‘systemic symptoms syndrome’ OR ‘Hypersensitivity’ OR ‘DRESS’. Additional sources, including reference lists of included studies and conference proceedings, were manually reviewed.

### Inclusion and exclusion criteria

Case reports and case analysis of patients with eosinophilia and systemic symptoms syndrome induced by minocycline were included as a preliminary study. Reviews, animal experimentation, mechanism studies, preclinical studies, repeated cases, and studies with insufficient data, non-minocycline drug exposures, or the wrong population were excluded.

### Diagnostic criteria for DRESS

The most widely used diagnostic criteria of DRESS are contained in the Registry of Severe Cutaneous Adverse Reactions (RegiSCAR) scoring system (Kardaun et al., 2007; Calle et al., 2023), based on the following key clinical manifestations: (1) Fever with a core temperature >101.3°F (38.5°C), or an axillary temperature >100.4°F (38°C), (2) Enlarged lymph nodes in at least 2 different body sites, (3) Patients with eosinophilia and heterogeneous lymphocytes. Heterogeneous lymphocytes refer to a mixed population of lymphocyte subsets (including reactive/atypical forms), as defined by RegiSCAR criteria. (4) Skin involvement (extent of rash, whether suggestive of DRESS, biopsy), (5) Organ damage, e.g., elevated liver enzymes defined as either: Alanine transaminase (ALT) or conjugated bilirubin at least two-fold over the upper normal limit on at least two separate dates, or Aspartate transaminase (AST), total bilirubin, and alkaline phosphatase (AP) all at least two-fold over the upper normal limit at least once (Kardaun et al., 2014), (6) Duration of illness >15 days. Each clinical manifestation was scored from −1 to 2 points based on severity, with total scores categorizing diagnostic certainty as negative case (<2 points), possible case (2-3 points), probable case (4-5 points), or definitive case (>5 points). To ensure diagnostic consistency, two independent reviewers applied the RegiSCAR criteria to all included cases. Discrepancies were resolved via consensus or referral to a senior dermatologist with expertise in hypersensitivity reactions. Studies reporting incomplete diagnostic data were excluded.

### Data extraction

Based on inclusion and exclusion criteria, studies were selected independently according to the diagnostic criteria by two researchers. Disagreements were resolved through consensus or referral to a senior researcher when needed. A specially designed data extraction table was used to extract the following information of the patients: nationality, sex, age, primary disease, primary indications, time of symptom onset, clinical symptoms, laboratory investigation, imaging examination, therapy, time of symptom improvement, clinical outcome, hospitalization days, and follow-up time.

### Statistical analysis

Statistical analyses were processed using IBM SPSS Statistics for Windows, Version 26.0. For continuous variables, if they conformed to a normal distribution, the variables were presented as means ± standard deviation (SD), otherwise, they were presented in the form of median and interquartile ranges (IQR). Categorical variables were reported in the form of counts and percentages.

## Results

### Demographic information

**FIGURE 1:**
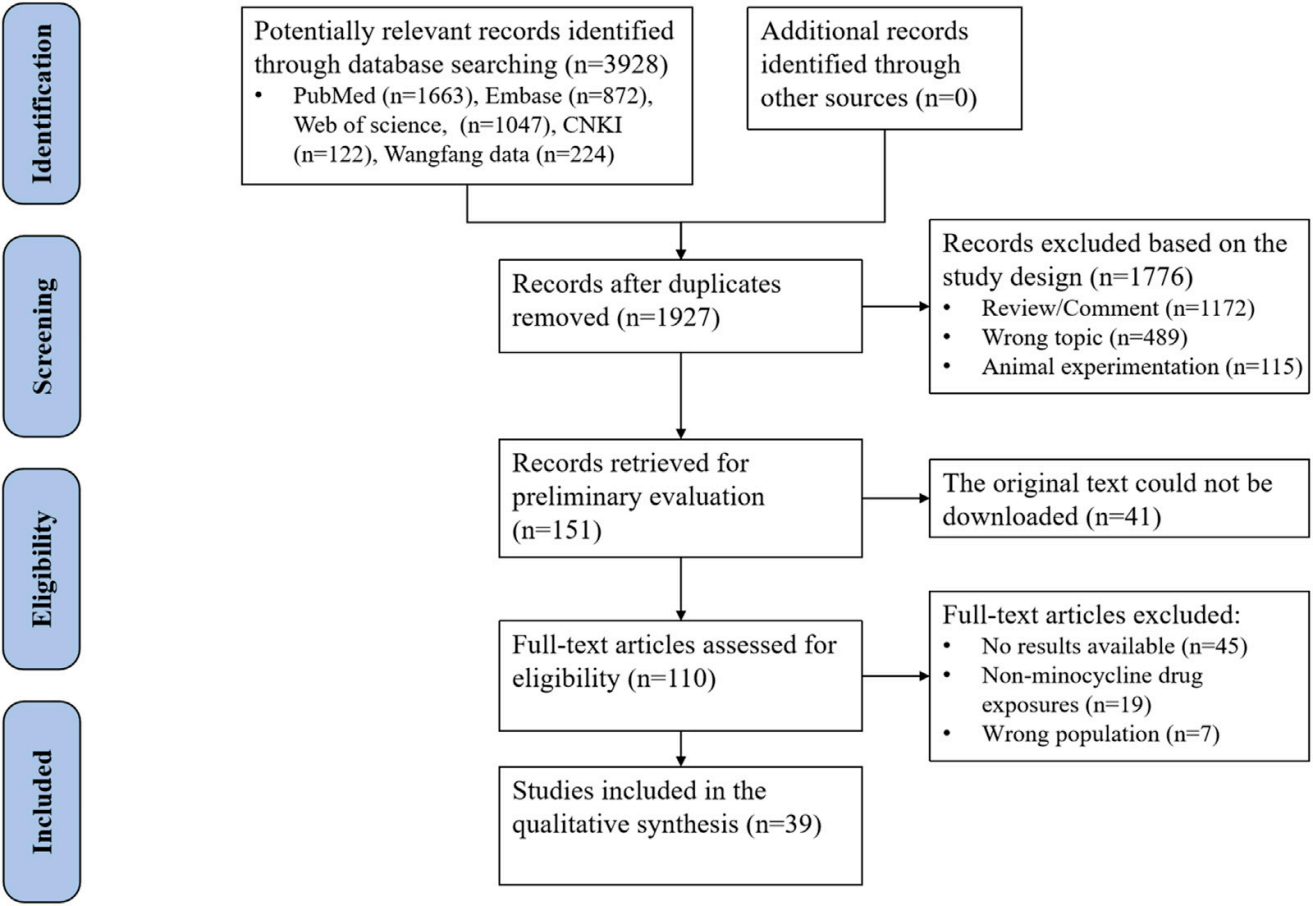
Screening flowchart for inclusion of studies.

In total, 3,928 citations were identified through a database search. After duplicate records and records that were not meeting the eligibility criteria were removed, a total of 39 case reports (comprising 57 patients), with a median age of 33 years, were included (Figure 1). Supplementary Table S1 systematically summarizes the clinical and diagnostic findings of the included DRESS cases, aligned with the RegiSCAR criteria. Table 1 shows detailed characteristics of the included individuals. The patients were mainly from Asia (19 cases, 33%), Europe (18 cases, 31.6%), America (15 cases, 26.3%), and Africa (two cases, 3.5%). Minocycline was mainly used to treat skin and soft tissue infections (40 cases, 71.4%) and respiratory infections (eight cases, 14.3%). Daily doses of minocycline ranged from 100 to 250 mg/day. The median medication time was 14 days (range, 7–24.8). The median days until symptom onset was 17.5 days (range, 7–25.8). Nine patients (29%) were transferred to the ICU. In the past medical history, 33.3% of 15 patients had respiratory disease. 26.7% of 15 patients had cardiovascular disease.

**TABLE 1:** Characteristics of the 57 included patients.

Parameter	Subcategory	Value/n (%)
Sex (n = 57)	Female	42 (73.7)
	Male	15 (26.3)
Age (n = 57)^a^	Years	33 (18.5–52)
Region (n = 54)	Asia (Japan 12, Caucasus 3, China 2, India 2)	19 (33.3)
	Europe (France 10, UK 2, Switzerland 2, Belgium 1, Israel 1, Netherlands 1, Spain 1)	18 (31.6)
	America (USA 14, Mexico 1)	15 (26.3)
	Africa (Guinea 1, Unknown 1)	2 (3.5)
	Unknown	3 (5.3)
Past medical history (n = 15)	Respiratory disease (Asthma 2, Allergic rhinitis 3)	5 (33.3)
	Cardiovascular disease (Hypertension 2, Coronary artery disease 1, Mitral valve prolapse 1)	4 (26.7)
	Endocrine system diseases (Obesity 1, Osteoporosis 1, Hyperthyroidism 1)	3 (20)
	Mental diseases (Attention deficit hyperactivity disorder and bipolar disorder 1, Major depressive disorder 1)	2 (13.3)
	Fibroids	1 (6.7)
	Glaucoma	1 (6.7)
	Rash	1 (6.7)
	Raynaud’s syndrome	1 (6.7)
	Undifferentiated connective tissue disease	1 (6.7)
	Interstitial granulomatous dermatitis	1 (6.7)
	Cystitis	1 (6.7)
Indications for minocycline use (n = 56)		
Infections		54 (96.4)
Skin and soft tissue infections	Facial acne 32, Furuncle 1, Cellulitis 1, Folliculitis 1, Papillomatosis 1, Rash 1, Perioral dermatitis 1, Cutaneous Mycobacterium marinum infection 1, Central centrifugal cicatricial alopecia 1	40 (71.4)
Respiratory infections	Pharyngitis 4, Pneumonia 3, Bronchitis 1	8 (14.3)
Urinary tract infections	Ureaplasma urealyticum gynecologic infection 3, Urethral burns 1	4 (7.1)
Brucella infectious disease		1 (1.8)
Lyme disease		1 (1.8)
Swelling in lower limbs		1 (1.8)
Fever & Cough		1 (1.8)
Medication time of minocycline (n = 52)^a^	Days	14 (7–24.8)
	1∼10	22 (42.3)
	11∼20	8 (15.4)
	21∼30	17 (32.7)
	>30	5 (9.6)
Days until symptom onset (n = 42)^a^	Days	17.5 (7–25.8)
	1∼10	14 (33.3)
	11∼20	7 (16.7)
	21∼30	18 (42.9)
	>30	3 (7.1)
Transferred to the ICU (n = 31)	Yes	9 (29)
	No	22 (71)

^a^ Median (interquartile ranges).

Data are n (%) unless otherwise indicated.

### Clinical manifestations of drug reactions

The clinical manifestations are summarized in Table 2. Vital signs included a mean temperature of 39.1°C in 26 patients. Symptoms (50 cases, 87.7%) included non-productive cough, dyspnea, pharyngitis, and wheezing were prevalent. Forty-five (78.9%) patients had the symptom of fever, and rash was observed in 17 patients (29.8%). Musculoskeletal and regional pain symptoms were reported in 16 patients (28.1%), including myalgia (31.3%), chest pain (25%), arthralgia (18.8%), headache (12.5%), neck pain (6.3%), and back pain (6.3%). Lymphadenopathy was present in 12 patients (21.1%), with cervical adenopathy specifically noted in five patients (8.8%). Fatigue was reported in eight patients (14%), and gastrointestinal symptoms (vomiting, nausea, anorexia, diarrhea) were observed in six patients (10.5%). In terms of organ-specific involvement, we counted 23 patients with hepatic involvement and 6 patients with gastrointestinal involvement based on clinical symptoms and laboratory investigation results. To highlight the distinct clinical patterns of minocycline-induced DRESS, we compared its features with those of DRESS caused by anticonvulsants, sulfonamides, and allopurinol (Supplementary Table S2).

**TABLE 2:** Clinical information of the 57 included patients.

Parameter	Value/n (%)
**Temperature (n = 26)** ^b^	39.1 ± 1.0
Blood pressure (n = 11)^b^	
Systolic Blood pressure	108.9 ± 24.4
Diastolic Blood pressure	69.5 ± 10.1
Clinical symptoms (n = 57)	
**Respiratory symptoms**	**50 (87.7)**
Non-productive cough	24 (48)
Dyspnea	21 (42)
Pharyngitis	3 (6)
Wheezing	2 (4)
**Fever**	**45 (78.9)**
**Rash**	**17 (29.8)**
**Musculoskeletal and regional pain symptoms**	**16 (28.1)**
Myalgia	5 (31.3)
Chest pain	4 (25)
Arthralgia	3 (18.8)
Headache	2 (12.5)
Neck pain	1 (6.3)
Back pain	1 (6.3)
**Lymphadenopathy**	**12 (21.1)**
**Facial edema**	**9 (15.8)**
**Fatigue**	**8 (14)**
**Gastrointestinal symptoms**	**6 (10.5)**
Vomit	3 (50)
Nausea	1 (16.7)
Anorexia	1 (16.7)
Diarrheic	1 (16.7)
**Chills**	**6 (10.5)**
**Cervical adenopathy**	**5 (8.8)**
**Erythematous eruption**	**4 (7)**
**Splenomegaly**	**3 (5.3)**
**Nightsweats**	**3 (5.3)**
**Scalp crusting**	**1 (1.8)**
**Lethargy**	**1 (1.8)**
**Palpitations**	**1 (1.8)**
**Haemoptysis**	**1 (1.8)**
Laboratory investigation	
Leukocyte×10^9^/L (n = 38)^a^	18.1 (10.6–25.5)
Neutrophilia×10^9^/L (n = 11)^a^	13.3 (7.5–19.7)
Eosinophilia×10^9^/L (n1/n2 = abnormal/total)^a^	
Initial hospitalization reported (29/37)	1.7 (0.9–3.6)
Peak reported (16/16)	4.09 (1.6–12.9)
Lymphocytes×10^9^/L (n = 13)^a^	3.1 (1.4–7)
Monocytes×10^9^/L (n = 7)^a^	0.6 (0.4–1.1)
Erythrocyte sedimentation rate mm/h (n = 20)^a^	41 (24–95.8)
C - reactive protein mg/dL (n = 22)^a^	8.5 (3.36–15.6)
IgE U/mL (n = 17)^a^	627.1 (198.5–1,624)
Alanine transaminase U/L (n = 23)^a^	114 (32–229)
Aspartate transaminase U/L (n = 23)^a^	86 (35–256)
Lactate dehydrogenase U/L (n = 16)^a^	512 (346.8–1,039.5)
pO_2_ mmHg (n = 19)^a^	59 (51–66)
pCO_2_ mmHg (n = 9)^a^	34.3 (30.3–36.9)
Cultures of urine and blood (n = 18)	
Positive	2 (11.1)
Negative	16 (88.9)
Bronco - alveolar lavage (n = 13)^a^	
Percentage of Eosinophils (%)	22 (12.3–37.3)
Percentage of Neutrophils (%)	6 (1–10.3)
Percentage of Macrophages (%)	53 (40.3–66.5)
Transbronchial lung biopsy	
Eosinophilic infltration (n = 14)	13 (92.9)
CD4^+^/CD8^+^ ratio (n = 9)^a^ (%)	1 (0.5–1.8)

^a^ Median (interquartile ranges).

^b^ Mean ± standard deviation.

Data are n (%) unless otherwise indicated.

Bold values represent primary categories for clarity in data presentation.

### Laboratory investigation

The laboratory test results are summarized in Table 2. Leukocytes were elevated in 38 (66.7%) of 57 patients, with a median value of 18.1*10^9^/L (range, 10.6–25.5). Neutrophilia occurred in 11 patients, with a median value of 13.3*10^9^/L (range, 7.5–19.7). Thirty-seven patients reported eosinophil results on the first day of admission, with a median value of 1.7*10^9^/L (range 0.9–3.6). Eosinophilia (>0.7*10^9^/L) was present in 78.4% (29/37) of patients at admission. The 16 patients who reported peak eosinophils during hospitalisation all had abnormal values with a median of 4.09*10^9^/L (range 1.6–12.9). Lymphocyte values ranged from 1.4 to 7 *10^9^/L, with a median of 3.1*10^9^/L. Seven patients reported monocytes value with a median value of 0.6*10^9^/L (range, 0.4–1.1). Urine and blood cultures were provided by 18 patients to rule out concurrent infections, particularly in febrile patients. Positive cultures were identified in 2 cases (11.1%). These results guided adjunctive antimicrobial therapy but did not alter the primary diagnosis of DRESS, which was confirmed by RegiSCAR criteria and histopathology. The erythrocyte sedimentation rate (ESR) was elevated in 20 patients (35.1%), with a median value of 41 mm/h (range, 24–95.8). The median C-reactive protein level was 8.5 mg/dL (range, 3.36–15.6). IgE was reported in 17 patients, with a median value of 627.1U/mL (range, 198.5–1,624). Twenty-three patients provided alanine transaminase and aspartate transaminase, with a median value of 114U/L (range, 32–229) and 86U/L (range, 35–256), respectively. Lactate dehydrogenase was elevated in 16 patients (28.1%), with a median value of 512U/L (range, 346.8–1,039.5). The broncho-alveolar lavage results from 13 patients showed that the percentage of eosinophils was 22% (interquartile range 12.3%–37.3%), the percentage of neutrophils was 6% (interquartile range 1%–10.3%), and the percentage of macrophages was 53% (interquartile range 40.3%–66.5%). Additionally, transbronchial lung biopsy results from 14 patients revealed eosinophilic infiltration in 13 cases (92.9%). These findings highlight a significant presence of eosinophils in both broncho-alveolar lavage fluid and lung tissue, indicating a potential eosinophilic inflammatory process in the included patients.

### Imaging examination

The imaging examination results are summarized in Table 3. The imaging examination results of the 57 included patients showed that among chest radiograph findings (n = 35), 14.3% were normal, while abnormal findings included interstitial lung changes (14.3%), ground glass opacity (22.9%), pleural effusion (31.4%), reticulonodular opacities (5.7%), micronodules (5.7%), diffuse reticular shadow (2.9%), infiltration (31.4%), inflammatory changes (2.9%), hyperdense shadow (2.9%), and mediastinal adenopathies (2.9%). Among chest computed tomography findings (n = 21), 9.5% were normal, while abnormal findings included ground glass opacity (42.9%), diffuse interstitial shadows (19%), air space consolidation (19%), pleural effusion (28.6%), lymphadenopathy (14.3%), bronchial wall thickening (23.8%), bronchitis (4.8%), and interlobular septal thickening (4.8%).

**TABLE 3:** Pulmonary imaging examination of the 57 included patients.

Parameter	Chest radiograph (n = 35)	Chest computed tomography (n = 21)
Normal	5 (14.3)	2 (9.5)
Abnormal Findings		
Interstitial Lung Abnormalities		
Ground glass opacity	8 (22.9)	9 (42.9)
Diffuse interstitial shadows	—	4 (19)
Interstitial lung changes	5 (14.3)	—
Reticulonodular opacities	2 (5.7)	—
Micronodules	2 (5.7)	—
Diffuse reticular shadow	1 (2.9)	—
Alveolar/Parenchymal Abnormalities		
Infiltration	11 (31.4)	3 (14.3)
Inflammatory changes	1 (2.9)	-
Hyperdense shadow	1 (2.9)	-
Air space consolidation	—	4 (19)
Pleural/Pericardial Abnormalities		
Pleural effusion	11 (31.4)	6 (28.6)
Lymphadenopathy		
Lymphadenopathy	—	3 (14.3)
Mediastinal adenopathies	1 (2.9)	—
Airway Abnormalities		
Bronchial wall thickening	—	5 (23.8)
Bronchitis	—	1 (4.8)
Miscellaneous		
Kerley B line	7 (20)	—
Interlobular septal thickening	—	1 (4.8)

—, unknown.

### Treatment and outcomes

The results are summarized in Table 4. After patients developed DRESS, 54 patients reported treatment outcomes, and all discontinued minocycline. Thirty-six patients (66.7%) received steroid treatment, including intravenous or oral methylprednisolone, dexamethasone, or prednisone. Six (11.1%) patients needed mechanical ventilatory support. The median time of symptom improvement was 10.5 days (range, 3–15), and 11 (37.9%) cases had improvement in clinical symptoms in 1–7 days, 11 (37.9%) cases in 8–14 days, 3 (10.3%) cases in 15–21 days, and 4 (13.8%) cases in more than 21 days. Minocycline provocation test was done in 14 patients after discontinuation of minocycline, of which 57.1% (8 cases) came back positive and 42.9% (6 cases) negative. Six patients reported undergoing lymphocyte transformation testing, of which 2 (33.3%) tested positive and four (66.7%) tested negative. Eventually, a total of 45 patients (93.8%) recovered after treatment. Unfortunately, 4 patients died due to recalcitrant hypotension and asystole (1 case), hepatic failure (1 case), DRESS (1 case), and unknown cause (1 case). Hospitalization days were recorded in 11 patients: 9 (81.8%) within 30 days, 2 (18.2%) more than 30 days, and the median time of hospitalization days was 15 days (range, 3.8–25). Follow-up data were available for 11 patients, with a median follow-up duration of 6 months (range: 1–12 months). Among these, 4 patients (36.4%) were followed for less than 3 months, and seven patients (63.6%) were followed for more than 3 months.

**TABLE 4:** Treatment and prognosis of the 57 included patients.

Parameter	Subcategory	Value/n (%)
Interventions for managing minocycline-associated DRESS syndrome (n = 54)	Discontinued minocycline	54 (100)
	Steroids use	36 (66.7)
	Mechanical ventilatory support	6 (11.1)
Time of symptom improvement (n = 29)^a^	Days	10.5 (3–15)
	1∼7	11 (37.9)
	8∼14	11 (37.9)
	15∼21	3 (10.3)
	>21	4 (13.8)
Minocycline provocation test (n = 14)	Positive	8 (57.1)
	Negtive	6 (42.9)
Lymphocyte transformation test (n = 6)	Positive	2 (33.3)
	Negtive	4 (66.7)
Clinical outcome (n = 48)		
Recover		45 (93.8)
Death		4 (8.3)
Cause of death (n = 4)		
DRESS		1 (25)
Recalcitrant hypotension and asystole		1 (25)
Acute hepatic failure		1 (25)
Unknown		1 (25)
Hospitalization days (n = 11)^a^	Days	15 (3.8–25)
	<30	9 (81.8)
	>30	2 (18.2)
Follow-up time (n = 11)^a^	Months	6 (1–12)
	<3	4 (36.4)
	>3	7 (63.6)

^a^ Median (interquartile ranges).

Data are n (%) unless otherwise indicated.

## Discussion

Drug reaction with eosinophilia and systemic symptoms (DRESS) is a syndrome of drug-induced hypersensitivity reactions, characterized primarily by rash, fever, hematologic and visceral organ involvement (Wei et al., 2024). First reported to be triggered by aromatic anticonvulsants, it has been successively reported that antibiotics and allopurinol also cause DRESS (Husain et al., 2013). The lack of reliable epidemiologic information on disease incidence and associated etiology has resulted in the absence of definitive studies counting the incidence of DRESS. Minocycline is a semi-synthetic tetracycline broad-spectrum antibiotic mainly used for pneumonia, acute bronchitis, urinary tract infections, respiratory tract infections, and acne due to *staphylococcus*, *streptococcus*, pneumococcus, and other pathogenic bacteria (Baldwin and Ward, 2021). Several adverse reactions, including life-threatening ones, have been reported in association with minocycline (Dominic, 2021). According to the French Pharmacovigilance Database (FPD), based on marketing authorization holders (MAH) data and published reports, minocycline-associated ADRs were more serious and were reported more frequently than for the other tetracyclines, especially autoimmune disorders, DRESS, and other hypersensitivity reactions (Lebrun-Vignes et al., 2012). This can prolong a patient’s medication regimen and hospitalization, increase medical costs, and even endanger a patient’s life. Therefore, we conducted this study to summarize the clinical features and outcomes of DRESS caused by minocycline.

The clinical manifestations of DRESS are not limited to eosinophilia, but also fever, cough, and pleurisy. Our finding that respiratory symptoms were the most common clinical manifestation (87.7%) aligns with the focused analysis by Taweesedt et al. which reported pulmonary involvement in 72% of definitive DRESS cases with lung involvement (Taweesedt et al., 2019). Notably, their study highlighted that pulmonary manifestations are less frequent in DRESS overall, underscoring that our higher prevalence may reflect minocycline-specific mechanisms (e.g., tissue accumulation) or a selection bias toward severe cases captured in published reports. However, our study observed a higher proportion of respiratory symptoms compared to the 50% interstitial infiltrates and 31% acute respiratory distress syndrome (ARDS) reported in that review. This discrepancy may be attributed to differences in study populations (pediatric vs. adult focus) or diagnostic criteria (RegiSCAR score ≥6 vs. clinical diagnosis). These similarities underscore the importance of maintaining a high index of suspicion for DRESS in patients presenting with unexplained respiratory symptoms and peripheral eosinophilia. Some patients are also accompanied by abnormally elevated CRP and ESR, and are therefore easily misdiagnosed. Therefore, it needs to be differentiated from other severe skin rashes, viral or bacterial infections, eosinophilia, lymphomas, and connective tissue diseases. Histopathology can specifically diagnose DRESS and monitor disease progression and response to treatment. Histopathology can support the differential diagnosis of DRESS by excluding alternative diseases, such as infections, lymphomas, or connective tissue disorders. However, it cannot definitively diagnose DRESS, as no pathognomonic histological features have been described for DRESS in skin or lung biopsies. In this study, transbronchial lung biopsy revealed eosinophilic infiltration in 13 of 14 cases (92.9%), suggesting a significant eosinophilic inflammatory process in the lungs of these individuals. While nonspecific, it aligns with the immune-mediated eosinophilic inflammation characteristic of DRESS and helps corroborate the clinical diagnosis when combined with other RegiSCAR criteria. The high prevalence of pulmonary involvement may reflect both the systemic distribution of minocycline and the immune-mediated eosinophilic infiltration observed in lung tissue (92.9% of biopsies). This aligns with the broader pathophysiology of DRESS, where drug-specific immune responses contribute to multi-organ inflammation, including the respiratory system.

The exact pathogenesis of DRESS is unknown. It is currently proposed that drug metabolism, immune dysregulation, and genetic susceptibility interact to trigger a systemic hypersensitivity response, which may be exacerbated by viral reactivation (e.g., herpesviruses) in susceptible individuals. However, drug exposure remains the primary and essential trigger for DRESS (Kardaun et al., 2013; Yamada et al., 2019). Minocycline is mainly metabolized by the liver and kidneys, with a plasma half-life of about 18 h (Saivin and Houin, 1988; Nelis and De Leenheer, 1982). Due to its high lipid solubility, minocycline easily penetrates into lipophilic tissues and body fluids, and is widely distributed in the body, especially prone to cause thyroid pigmentation and skin pigmentation (Jonas and Cunha, 1982). Eve Maubec et al. showed that minocycline accumulation in patients, especially in the skin, is associated with a prolonged course of DRESS (Maubec et al., 2008). This may mediate an autoimmune response that is ultimately a key factor in the poor clinical outcomes of DRESS. The median time to symptom improvement was 10.5 days, with 24.1% of patients (10.3% in 15–21 days and 13.8% in >21 days) requiring more than 2 weeks to achieve clinical recovery. Some studies have already shown, by positive patch tests and *in vitro* lymphocyte proliferation assays, that a strong drug-specific immune response is a key factor in the pathogenesis of DRESS (Genin et al., 2014; Barbaud et al., 2013). In our study, we reported two cases with positive results of the minocycline lymphocyte transformation test. Fourteen additional patients reported results of the minocycline provocation test, of which 57.1% were positive. We retained 6 cases with negative drug provocation tests results. This is because all 6 cases met the RegiSCAR criteria for a definite or probable diagnosis of DRESS based on clinical presentation, laboratory findings, and temporal association with minocycline exposure. In addition, we performed a sensitivity analysis to exclude these cases and found no substantial changes in our main findings regarding clinical features or prognosis. Our study reported below-normal CD4+/CD8+ ratios in nine patients. It has been shown that during the acute phase of DRESS, activated cytotoxic T cells (CD8^+^ T cells) and helper T cells (CD4^+^ T cells) expand, and the CD4+/CD8+ ratio usually decreases, which corroborates our results (Miyagawa et al., 2020). It has been established that individuals carrying specific mutations in genes that encode drug detoxification enzymes have a higher risk of DRESS (Tas and Simonart, 1999). Unlike other cyclines, for example, minocycline is metabolized in the liver, and the patients included in our study generally had abnormal liver function. Liver function tests (ALT/AST) were measured after DRESS symptom onset and showed median elevations of 114 U/L and 86 U/L, respectively. The observed transaminase elevations may result from a combination of minocycline’s hepatotoxic potential and immune-mediated liver injury, a hallmark of DRESS. While hepatic detoxification pathways could play a role, our study lacks direct evidence to confirm this mechanism. Unfortunately, our study did not report results regarding pharmacogenetic susceptibility.

Discontinuation of minocycline is an imperative step in managing suspected DRESS, as ongoing exposure risks exacerbating organ damage and mortality, as stipulated in the RegiSCAR diagnostic framework. The patients included in this study were treated with timely interventions to discontinue the drug. Treatment requires different interventions depending on the patient’s symptoms, including, but not limited to, antipyretic, cough suppression, rehydration, maintenance of electrolyte balance, nutritional support, and liver protection. Prioritize the use of highly effective topical corticosteroids when necessary. Although only one death was due to DRESS, and 93.8% of patients had a favorable outcome, there were still two cases of death due to severe involvement of vital organs. This demonstrates the critical importance of frequent monitoring and follow-up for patients with severe or rapidly progressing disease. Two patients in our study had relapses after improvement on initial treatment, and the duration of relapse was 6 weeks. Therefore, it cannot be ruled out that patients may develop autoimmune diseases and related sequelae months or years after the end of the drug response (Chen et al., 2013).

Several limitations of this study should be mentioned. This is insufficient for a comprehensive assessment of the clinical features of minocycline-induced DRESS, especially the assessment of risk factors. Subsequent analysis of relevant risk factors and predictive modeling based on a large data platform is required. This was a retrospective study, and parameters such as time to onset of adverse reactions and time to improvement of the patient’s symptoms may bias the results due to factors such as the reporting person’s recording preferences. As the included studies were from different regions, the test indicator eligibility ranges and indicator units varied, which may cause some bias to the results after standardization and harmonization.

## Conclusion

In conclusion, we comprehensively delineated the clinical characteristics, diagnostic challenges, and treatment outcomes of minocycline-associated DRESS syndrome. The study underscores the importance of recognizing the diverse clinical manifestations, particularly respiratory symptoms, and the necessity of timely intervention through drug cessation and corticosteroid therapy. Despite the favorable prognosis in most cases, the potential for severe organ involvement and the risk of prolonged hospitalization highlights the critical need for vigilant monitoring and follow-up. Future research should focus on expanding the sample size and addressing the limitations of retrospective studies to further enhance our understanding of this complex syndrome and improve patient care.

## Data Availability

The raw data supporting the conclusions of this article will be made available by the authors upon reasonable request.
