# Supplementary material for: Clinical manifestations, diagnostic criteria, and treatment outcomes of minocycline-associated DRESS syndrome: a comprehensive exploration of published cases
Source: Front Pharmacol. 2025 Jul 22;16:1515000. doi: 10.3389/fphar.2025.1515000 (PMC12321776; doi:10.3389/fphar.2025.1515000)
Supplement: Supplementary file 1 [file Supplementaryfile2.doc]

**Table S2. Comparison of Clinical Features in Minocycline-Induced DRESS vs. Other Common Drug-Induced DRESS Syndromes (Anticonvulsants, Sulfonamides, Allopurinol)**

| ****Clinical Feature**** | ****Minocycline-Induced DRESS**** | ****Anticonvulsants (e.g., Carbamazepine)**** | ****Sulfonamides (e.g., TMP-SMX)**** | ****Allopurinol**** | ****References**** |
| --- | --- | --- | --- | --- | --- |
| ****Fever**** | Common (≥38°C) | Common (≥38°C) | Common (≥38°C) | Common (≥38°C) | 17, 22, 27 |
| ****Skin Rash**** | Maculopapular, often mild | Severe, exfoliative | Maculopapular, purpuric | Severe | 17, 20, 22 |
| ****Eosinophilia**** | Marked (often >1.5×10⁹/L) | Moderate to marked | Moderate | Marked | 17, 22, 30 |
| ****Lymphadenopathy**** | Cervical/common | Generalized | Localized | Rare | 17, 22 |
| ****Hepatic Involvement**** | Elevated ALT/AST | Hepatitis, cholestasis | Hepatitis | Fulminant hepatitis | 17, 22, 30 |
| ****Respiratory Symptoms**** | Dominant (cough, dyspnea) | Rare | Interstitial pneumonia | Rare | 15, 30 |
| ****Renal Involvement**** | Mild (acute tubular necrosis) | Interstitial nephritis | Acute kidney injury | Severe | 16, 22 |
| ****Cardiac Involvement**** | Myocarditis (rare) | Myocarditis/pericarditis | Rare | Rare | 17, 22 |
