# Supplementary material for: Clinical manifestations, diagnostic criteria, and treatment outcomes of minocycline-associated DRESS syndrome: a comprehensive exploration of published cases
Source: Front Pharmacol. 2025 Jul 22;16:1515000. doi: 10.3389/fphar.2025.1515000 (PMC12321776; doi:10.3389/fphar.2025.1515000)
Supplement: Supplementary file 2 [file Supplementaryfile1.doc]

**Table S1. Included Cases of DRESS diagnosed according to RegiSCAR criteria**

| **Case no.** | **Fever≥38.5℃** | **Enlarged lymph nodes** | **Eosinophilia** | **Atypical lymphocytes** | **Skin involvement** | **Organ involvement** | **Resolution≥15 days** | **Evaluation other potential causes** | **Total score** |
| --- | --- | --- | --- | --- | --- | --- | --- | --- | --- |
| 1 | 0 | 0 | 1 | 1 | 0 | 1 | 0 | 1 | 4 |
| 2 | 0 | 0 | 2 | 0 | 0 | 1 | 0 | 1 | 4 |
| 3 | -1 | 1 | 1 | 1 | 0 | 1 | 0 | 1 | 4 |
| 4 | 0 | 0 | 1 | 1 | 0 | 1 | 0 | 1 | 4 |
| 5 | 0 | 0 | 2 | 0 | 0 | 1 | 0 | 1 | 4 |
| 6 | -1 | 1 | 0 | 1 | 0 | 2 | 0 | 1 | 4 |
| 7 | 0 | 1 | 1 | 1 | 1 | 0 | 0 | 1 | 5 |
| 8 | 0 | 0 | 1 | 1 | 0 | 1 | 0 | 1 | 4 |
| 9 | 0 | 0 | 1 | 0 | 0 | 2 | 0 | 1 | 4 |
| 10 | 0 | 0 | 1 | 1 | 0 | 1 | 0 | 1 | 4 |
| 11 | 0 | 0 | 1 | 1 | 0 | 1 | 0 | 1 | 4 |
| 12 | 0 | 0 | 1 | 0 | 0 | 2 | 0 | 1 | 4 |
| 13 | 0 | 0 | 1 | 0 | 0 | 2 | 0 | 1 | 4 |
| 14 | 0 | 0 | 1 | 0 | 0 | 2 | 0 | 1 | 4 |
| 15 | 0 | 0 | 2 | 0 | 0 | 2 | -1 | 1 | 4 |
| 16 | -1 | 1 | 1 | 1 | 0 | 1 | 0 | 1 | 4 |
| 17 | 0 | 0 | 2 | 0 | 2 | 1 | -1 | 1 | 5 |
| 18 | -1 | 1 | 2 | 1 | 0 | 2 | -1 | 1 | 5 |
| 19 | 0 | 0 | 2 | 0 | 0 | 2 | 0 | 1 | 5 |
| 20 | 0 | 0 | 2 | 0 | 0 | 1 | 0 | 1 | 4 |
| 21 | -1 | 0 | 2 | 0 | 2 | 1 | -1 | 1 | 4 |
| 22 | 0 | 0 | 1 | 0 | 0 | 2 | 0 | 1 | 4 |
| 23 | 0 | 0 | 2 | 0 | 0 | 1 | 0 | 1 | 4 |
| 24 | 0 | 1 | 1 | 1 | 2 | 1 | 0 | 1 | 7 |
| 25 | 0 | 0 | 2 | 1 | 0 | 1 | -1 | 1 | 4 |
| 26 | -1 | 1 | 2 | 1 | 1 | 1 | -1 | 0 | 4 |
| 27 | 0 | 0 | 2 | 0 | 1 | 2 | -1 | 1 | 5 |
| 28 | 0 | 1 | 2 | 1 | 2 | 2 | 0 | 0 | 8 |
| 29 | 0 | 1 | 0 | 1 | 0 | 1 | 0 | 1 | 4 |
| 30 | 0 | 1 | 2 | 1 | 2 | 2 | 0 | 1 | 9 |
| 31 | 0 | 0 | 2 | 0 | 0 | 2 | 0 | 1 | 5 |
| 32 | -1 | 1 | 1 | 1 | 1 | 2 | 0 | 1 | 6 |
| 33 | 0 | 0 | 2 | 0 | 1 | 2 | 0 | 1 | 6 |
| 34 | 0 | 0 | 0 | 0 | 2 | 2 | 0 | 1 | 5 |
| 35 | 0 | 0 | 2 | 0 | 0 | 1 | 0 | 1 | 4 |
| 36 | 0 | 0 | 2 | 0 | 0 | 1 | 0 | 1 | 4 |
| 37 | 0 | 0 | 1 | 1 | 0 | 2 | 0 | 1 | 5 |
| 38 | 0 | 0 | 2 | 0 | 0 | 2 | 0 | 1 | 5 |
| 39 | 0 | 0 | 2 | 0 | 0 | 1 | 0 | 1 | 4 |
| 40 | 0 | 0 | 2 | 0 | 1 | 0 | 0 | 1 | 4 |
| 41 | 0 | 0 | 2 | 0 | 0 | 1 | 0 | 1 | 4 |
| 42 | -1 | 0 | 0 | 1 | 2 | 1 | 0 | 1 | 4 |
| 43 | -1 | 1 | 2 | 1 | 1 | 0 | 0 | 1 | 5 |
| 44 | 0 | 0 | 2 | 0 | 0 | 1 | 0 | 1 | 4 |
| 45 | 0 | 1 | 0 | 1 | 0 | 2 | 0 | 0 | 4 |
| 46 | -1 | 0 | 2 | 0 | 2 | 2 | -1 | 1 | 5 |
| 47 | 0 | 0 | 2 | 0 | 1 | 0 | 0 | 1 | 4 |
| 48 | 0 | 1 | 2 | 1 | 2 | 1 | 0 | 1 | 8 |
| 49 | 0 | 0 | 2 | 0 | 1 | 1 | -1 | 1 | 4 |
| 50 | 0 | 0 | 2 | 0 | 1 | 1 | -1 | 1 | 4 |
| 51 | -1 | 1 | 2 | 1 | 2 | 1 | 0 | 1 | 7 |
| 52 | -1 | 1 | 1 | 1 | 1 | 2 | -1 | 1 | 5 |
| 53 | -1 | 0 | 2 | 0 | 2 | 2 | -1 | 0 | 4 |
| 54 | 0 | 1 | 0 | 1 | 2 | 1 | -1 | 0 | 4 |
| 55 | 0 | 0 | 2 | 0 | 1 | 2 | -1 | 0 | 4 |
| 56 | -1 | 1 | 2 | 1 | 2 | 2 | 0 | 1 | 8 |
| 57 | -1 | 0 | 2 | 0 | 0 | 2 | 0 | 1 | 4 |

RegiSCAR DRESS validation score:

Final score ＜2: No case

Final score 2-3: Possible case

Final score 4-5: Probable case

Final score＞5: Definite case
